# Comparisons of physical activity and understanding of the importance of exercise according to dialysis modality in maintenance dialysis patients

**DOI:** 10.1038/s41598-021-00924-0

**Published:** 2021-11-02

**Authors:** Jun Chul Kim, Jun Young Do, Seok Hui Kang

**Affiliations:** 1 Division of Nephrology, Department of Internal Medicine, CHA Gumi Medical Center, CHA University, Gumi, Republic of Korea; 2 Department of Internal Medicine, Yeungnam University College of Medicine, 170, Hyeonchung-ro, Nam-gu, Daegu, 42415 Korea

**Keywords:** Health care, Nephrology

## Abstract

Data regarding the status of physical activity or understanding of the importance of exercise, such as barriers of exercise or enablers of exercise, in dialysis patients were insufficient. This study aimed to evaluate the status of physical activity and the understanding of the importance of exercise in Korean dialysis patients. The study participants were recruited from 27 hospitals or dialysis centers (n = 1611). Physical activity was evaluated using the Korean version of the International Physical Activity Questionnaire-Short Form. High physical activity was defined as ≥ 600 metabolic equivalent of task (MET). Knowledge about the importance of exercise, enabler for regular exercise, benefits of exercise, and barrier to exercise was evaluated. Health-related quality of life (HRQoL) was assessed by the Kidney Disease Quality of Life version 1.3. The number of participants in the hemodialysis (HD) and peritoneal dialysis (PD) groups was 1247 and 364, respectively. The intensity of physical activity did not differ between the two modalities. The time of physical activity was longer in HD patients than in PD patients, which resulted in greater MET values and the number of high physical activity. There were 762 (61.1%) HD patients and 281 (77.2%) PD patients who heard of the importance of exercise (*P* < 0.001). In both HD and PD patients, dialysis staff played the most significant role as educators on the importance of exercise and enablers of exercise. The most important barriers to exercise were poor motivation and fatigue in both modalities. HD patients exhibited greater differences in HRQoL scales across two physical activity levels, compared to PD patients. Our study showed that the barrier to exercise and the enablers of exercise were poor motivation/fatigue and encouragement from dialysis staff, respectively.

## Introduction

End-stage renal disease is an advanced stage of chronic kidney disease requiring renal replacement therapies such as hemodialysis (HD), peritoneal dialysis (PD), or kidney transplantation^1, 2^. Most end-stage renal disease patients have undergone dialysis because of organ shortage^1, 2^. High mortality and morbidity rates are observed in dialysis patients^3^. Advances in dialysis technologies can lead to improved long-term survival in dialysis patients. However, a uremic condition in dialysis patients is a well-known premature aging process^4^. Improvement in the long-term survival of dialysis patients increases exposure duration to a uremic condition, leading to an increased prevalence of physical disability^5^. This may result to another medical care burden.

It is well known that the prevalence of physical disability in dialysis patients is higher than that in the general population^6^. Therefore, knowledge about the importance of exercise to attenuate this problem has been addressed. Previous studies have evaluated knowledge about the importance of exercise in dialysis patients. A recent meta-analysis evaluated 21 randomized trials, and merged data showed that exercise improved the muscle mass and/or muscle strength^7^. However, all studies showed inconsistent results. Data regarding the current status of physical activity or understanding of the importance of exercise, such as barriers of exercise or enablers of exercise, in dialysis patients were insufficient. In addition, dialysis modality, region, or ethnicity can affect these associations, but there were insufficient data regarding dialysis modality and few data on Korean dialysis patients. This study aimed to evaluate the status of physical activity and the understanding of the importance of exercise in Korean dialysis patients.

## Patients and methods

### Study population

The study participants were initially enrolled in a previous study^8^. Briefly, the study participants were recruited from 27 hospitals or dialysis centers in Daegu/Kyungsangpook-do between July and December 2012. In total, 2737 participants who received dialysis were included. Participants were excluded if they met the following criteria: age < 20 years (n = 12), history of hospitalization during the past 3 months except for vascular access problems (n = 351), dialysis duration < 6 months (n = 164), unable to walk with or without an assistive device (n = 79), lack of laboratory findings (n = 117), refusal to participate (n = 254), or unable to communicate with interviewer (n = 149). Finally, 1611 participants were ultimately included. This study was approved by the institutional review board of a tertiary medical center (Approval number: 2016-06-022). Informed consent was obtained from all participants. The study was conducted in accordance with the principles of the Declaration of Helsinki.

### Study variables

The study variables have been previously described^8^. Briefly, demographic and laboratory data collected upon enrollment included the following: age, sex, body mass index (BMI; kg/m^2^), diabetes mellitus (DM), coronary artery disease (CAD), cerebrovascular disease (CVD), dialysis vintage (years), education level, and hemoglobin (mg/dL), blood urea nitrogen (BUN; mg/dL), creatinine (mg/dL), calcium (mg/dL), phosphorus (mg/dL), total cholesterol (mg/dL), serum potassium (mEq/L), intact parathyroid hormone (i-PTH; pg/mL), and high-sensitivity C-reactive protein (hs-CRP; mg/dL) levels, dialysis modality (HD or PD), and type of dialysis or access (arteriovenous fistula [AVF] or arteriovenous graft [AVG] in HD patients and continuous ambulatory PD [CAPD] or automated PD [APD] in PD patients).

DM was evaluated using three indicators: (1) a patient-reported history of DM, (2) the medical record of DM diagnosis or medication, and (3) a fasting glucose level ≥ 126 mg/dL. We evaluated all three indicators for all the patients and DM was diagnosed if one or more indicators among the three were positive. CAD was evaluated using two indicators: (1) a patient-reported history of angina, myocardial infarction, or congestive heart failure, and (2) the medical record for the presence of angina, myocardial infarction, or congestive heart failure. CVD was evaluated using two indicators: (1) a patient-reported history of stroke, and (2) the medical record for the presence of stroke. We evaluated all two sources of evidence for all patients and CAD or CVD were diagnosed if one or more among two indicators were positive.

### Assessment for status of physical activity and understanding of the importance of exercise

Physical activity was evaluated using the Korean version of the International Physical Activity Questionnaire-Short Form^9^. Briefly, physical activity was evaluated regardless if it was leisure or working. Total physical activity was calculated by metabolic equivalent of task (MET) values using the intensity and time of the physical activity. A 3.3 MET was identified for low-intensity physical activity such as walking, 4.0 MET for moderate-intensity physical activity such as moving lightweight materials or cycling in moderate speed, and 8.0 MET for vigorous-intensity physical activity such as aerobics, running, or cycling in high speed. Total MET values were calculated as the sum of MET values × time of physical activity (minutes/week). High physical activity was defined as ≥ 600 MET regardless of intensity or time of physical activity.

All participants were provided with the following questions: “Has the participant ever heard the importance of exercise?” The answer was either “yes” or “no.” If the participant answered “yes,” the next question was “Who told you of the importance of exercise?” The answers were physician, nurse, other dialysis patients, social or mass media, family, friend, nutritionist, social worker, or others. For all participants, the next question was “What is the enabler for regular exercise?” The answers were encouragement from physician; encouragement from nurse; encouragement from family, relatives, or friends; participation in a specialized exercise program; encouragement from other dialysis patients; or presence of an exercise facility.

Further questions were evaluated according to the performance of exercise. If the participant exercised, the question was “What are the benefits of exercise?” The answers were mood, strength, overall vitality (VT), sleeping, confidence, blood pressure, doing the things that the patient wants for himself/herself, muscle cramping, intradialytic hypotension, or others. If the participant did not exercise, the question was “What is the barrier to exercise?” The answers were poor motivation, fatigue, lack of time to exercise, pain, uncertain on how to exercise, depressive mood, fear of injury, lack of equipment or place to exercise, or unsure about the importance of exercise. The answers were in multiple choices.

### Definitions

We evaluated disability using four questions with regard to the activities of daily living to assess whether the participants currently needed help from another person to have a meal, dress/undress, get in or out of bed, or take a bath or shower. For each question, they answered one of three responses: “No help needed”; “Some help needed”; “Complete help needed”. Disability was defined as the inability to perform at least one of the four activities of daily living domains without help^10^.

Exhaustion was measured using two questions from the Center for Epidemiological Studies Depression (CES-D) Scale^11^: (1) I felt that everything I did was an effort and (2) I could not get going. Then the following question was asked: “How often did you feel this way?” The answer was rarely or none of the time, some or a little of the time, a moderate amount of the time, or most of the time. The participants answering moderate or most of the time to either or both of the two questions were identified as those experiencing exhaustion. A fall was defined as an event that resulted in a participant coming to rest unintentionally on the ground or a lower level with or without losing consciousness during the last 12 months^12^.

Frailty was defined using the modified criteria previously described elsewhere^10, 13–15^. Components included slowness, poor endurance, physical inactivity, and unintentional weight loss. Slowness and poor endurance were determined by the physical functioning (PF) scale of the Short-Form-36 (SF-36) (2 points for PF scale < 75) and the VT scale of the SF-36 (1 point for VT scale < 55), respectively. Participants who were physically inactive were defined as inactive group (1 point for physical inactivity). Unintentional weight loss was defined as unintentional body weight loss > 4.5 kg or 5% of the baseline value over the past year (1 point for weight loss). All points of scoring of each frailty component were summed. Those participants who scored with ≥ 3 points were defined as having frailty. Cut-off values for PF, VT, or weight loss were defined from the Cardiovascular Health Study, Women’s Health Initiative Observational Study, and the Dialysis Morbidity and Mortality Study Wave 2^13–15^. These studies diagnosed frailty using items on the SF-36. Previous studies used a cut-off value dichotomized at the 25th percentile of PF or VT. The cut-off of PF was highly associated with slow walking speed and that of VT was associated with poor endurance. Therefore, 75 < PF and 55 < VT were used to identify slowness and poor endurance, respectively. In addition, the Cardiovascular Health Study and Women’s Health Initiative Observational Study defined unintentional weight loss as the loss of > 10 pound (4.5 kg) over the past year, or more than 5% of the body weight in the previous 2 years. Therefore, we used a similar cut-off value for unintentional weight loss.

### Assessment of health-related quality of life scale scores

Health-related quality of life (HRQoL) was assessed by the Kidney Disease Quality of Life (KDQOL) version 1.3 Korean version^16^. KDQOL version 1.3 includes the SF-36 scale (36 items) and kidney disease-specific scale (11 items). The SF-36 scale includes the following eight domains: PF, role limitations due to physical health problems (RP), body pain, general health, VT, social functioning, role limitations due to emotional problems (RE), mental health (MH), and overall health rating. A total score of 0–100 is calculated for each domain. A low score means a low HRQoL. These scales have been used to calculate the physical component scale (PCS) and mental component scale (MCS)^17, 18^. The 11-item kidney disease-specific scale consists of symptoms/problems, kidney disease effects, kidney disease burden, work status, cognitive function, quality of social interaction (QSI), sexual function, sleep, social support, patient satisfaction, and dialysis staff encouragement.

### Statistical analyses

Data were analyzed using the statistical software SPSS version 25 (Chicago, IL, USA). Categorical variables were expressed as both counts and percentages. Continuous variables were evaluated for the distributional assumption of normality using Kolmogorov–Smirnov test. Continuous variables are expressed as mean ± standard deviation for those with normal distribution and median (interquartile range) for those with non-normal distribution. For continuous variables with normal distribution, means were compared using Student’s *t*-test. For continuous variables with non-normal distribution, values were compared using Kolmogorov–Smirnov z test. Categorical data was expressed as numbers (percentage) and Pearson’s χ^2^ or Fisher’s exact test was used to analyze categorical variables. Logistic regression analysis was performed to identify the impact of the dialysis modality for high physical activity. Multivariate analysis was performed using the analysis of covariance or logistic regression analysis and adjusted for age; sex; BMI; DM; CAD; CVD; serum albumin, BUN, creatinine, total cholesterol, i-PTH, and hs-CRP levels; and dialysis modality (PD or HD) or type of dialysis or access (AVF, AVG, or PD). The level of statistical significance was set at *P* < 0.05.

## Results

### Participants’ characteristics

The number of participants in the HD and PD groups was 1247 and 364, respectively (Table 1). The mean age in the HD and PD groups was 56.4 ± 13.2 and 54.1 ± 11.9 years, respectively. HD patients were older than PD patients. BMI was greater in PD patients than in HD patients. The proportion of patients with DM, CAD, or CVD was greater in HD patients than in PD patients. The levels of albumin, BUN, or hs-CRP were greater in HD patients than in PD patients, while those of creatinine, total cholesterol, or i-PTH were greater in PD patients than in HD patients.

**Table 1:** Participants’ clinical characteristics.

	Total (n = 1611)	HD (n = 1247)	PD (n = 364)	*P* value
Age (years)	55.9 ± 12.9	56.4 ± 13.2	54.1 ± 11.9	0.003
Sex (male, %)	900 (55.9%)	705 (56.5%)	195 (53.6%)	0.316
Body mass index (kg/m^2^)	22.4 ± 3.2	22.1 ± 3.2	23.5 ± 3.1	< 0.001
Diabetes mellitus (%)	637 (39.5%)	514 (41.2%)	123 (33.8%)	0.011
Coronary artery disease (%)	254 (15.8%)	221 (17.7%)	33 (9.1%)	< 0.001
Cerebrovascular disease (%)	144 (8.9%)	129 (10.3%)	15 (4.1%)	< 0.001
Dialysis vintage (years)	5.1 ± 4.5	5.1 ± 4.6	5.3 ± 3.9	0.422
**Education level**				0.114
≤ 6th grade	353 (21.9%)	285 (22.9%)	68 (18.7%)	
7th to 12th grade	318 (19.7%)	251 (20.1%)	67 (18.4%)	
> 12th grade	940 (58.3%)	711 (57.0%)	229 (62.9%)	
Hemoglobin (mg/dL)	10.5 (0.9)	10.5 (0.7)	10.4 (1.2)	0.063
Serum albumin (g/dL)	3.9 ± 0.4	4.0 ± 0.3	3.6 ± 0.4	< 0.001
Blood urea nitrogen (mg/dL)	57.6 (21.1)	61.7 (19.5)	51.1 (19.2)	< 0.001
Creatinine (mg/dL)	10.5 ± 3.0	10.3 ± 2.8	11.1 ± 3.6	< 0.001
Calcium (mg/dL)	8.7 ± 0.8	8.7 ± 0.8	8.6 ± 0.9	0.076
Phosphorus (mg/dL)	5.3 ± 1.4	5.3 ± 1.4	5.3 ± 1.3	0.667
Total cholesterol (mg/dL)	152.0 (48.0)	140.5 (45.0)	171.5 (44.5)	< 0.001
Intact parathyroid hormone (pg/mL)	188.2 (232.2)	179.3 (207.9)	215.1 (268.2)	0.042
High sensitivity C-reactive protein (mg/dL)	0.16 (0.46)	0.21 (0.53)	0.09 (0.32)	< 0.001

Data are expressed as numbers (percentages) for categorical variables and means ± standard deviations for continuous variables with normal distribution, and median (interquartile range) for continuous variables without normal distribution. *P-*values were tested by Student’s *t*-test or Kolmogorov–Smirnov z test for continuous variables and Pearson’s χ^2^ or Fisher’s exact tests for categorical variables. *P*-values were calculated between the HD and PD groups. Abbreviations: HD, hemodialysis; PD, peritoneal dialysis.

### Current status of physical activity according to dialysis modality

The number of patients with no, low-, moderate-, or vigorous-intensity physical activity was 567 (45.5%), 427 (34.2%), 219 (17.6%), and 34 (2.7%) in HD patients and 161 (44.2%), 135 (37.1%), 63 (17.3%), and 5 (1.4%) in PD patients, respectively (*P* = 0.409). The time of physical activity was 30 (120) minutes in HD and 30 (87.5) minutes in PD patients, respectively (*P* = 0.014). The MET value was 90 (360) in HD and 90 (270) in PD patients, respectively (*P* = 0.021). The two groups had different distributions in time of physical activity and MET. The number of HD or PD patients with high physical activity was 140 (11.2%) and 20 (5.5%), respectively (P = 0.001). The proportion of patients with high MET was greater in HD patients than in PD patients. Multivariate analysis-adjusted covariates showed that MET values in HD and PD patients were 325.1 ± 1.1 and 241.8 ± 1.1, respectively (*P* = 0.002). Multivariate linear regression analyses showed that old age, female sex, and high BMI levels were inversely associated with MET levels (Table S1). High BUN levels and HD modality were positively associated with MET level.

Logistic regression analysis showed that the odds ratio for high physical activity in HD patients was 2.18 (1.34–3.53) compared to that in PD patients (Table S2, *P* = 0.002). The odds ratio for high physical activity in HD patients was 3.01 (1.64–5.54) compared to that in PD patients on multivariate analysis (*P* < 0.001). In addition, multivariate logistic regression analysis showed that those are old in age was associated with lower odds ratio for high physical activity. Those that have high serum albumin, creatinine, or total cholesterol, and undergo HD, were associated with higher odds ratio for high physical activity.

The numbers of patients with disability, frailty, fall, or exhaustion were 195 (15.6%) in HD and 109 (29.9%) in PD (*P* < 0.001), 421 (33.8%) in HD and 136 (37.4%) in PD (*P* = 0.204), 225 (18.0%) in HD and 60 (16.5%) in PD (*P* = 0.493), and 347 (27.8%) in HD and 124 (34.1%) in PD (*P* = 0.021), respectively. Table S3 show the proportions of patients with disability, frailty, fall, or exhaustion according to physical activity. The participants without high physical activity had significantly higher prevalence of disability, faulty, or exhaustion in HD patients and frailty in PD patients compared to those with high physical activity. Although statistical significance was not obtained in fall in HD patients and disability, fall, or exhaustion in PD patients, the trends were similar.

The numbers of PD patients who had undergone CAPD or APD were 196 (53.8%) and 89 (24.5%), respectively. There were no data for PD modality in 79 patients (21.7%). The numbers with CAPD or APD performing high physical activity were 9 (4.6%) and 6 (6.7%), respectively (*P* = 0.451). MET values in CAPD or APD patients were 90 (270) and 90 (310), respectively (*P* = 0.385). There was no significant difference in the proportion of high physical activity and MET values between CAPD and APD.

### Patients’ understanding of the importance of exercise according to dialysis modality

There were 762 (61.1%) HD patients and 281 (77.2%) PD patients who heard of the importance of exercise (*P* < 0.001). In both HD and PD patients, dialysis staff (physician and/or nurse) played the most significant role as educators on the importance of exercise and enablers of exercise (Table 2). The most commonly selected benefits from exercise were mood, strength, and overall VT in both HD and PD patients. Poor motivation and fatigue were the most common barriers to exercise in both HD and PD patients.

**Table 2:** Comparison of exercise-related questionnaires according to dialysis modality.

	Total	HD	PD	*P* value
**Education for the importance of exercise**				< 0.001
Physician	519 (24.7%)	346 (24.2%)	173 (25.7%)	
Nurse	559 (26.6%)	349 (24.4%)	210 (31.3%)	
Other dialysis patient	296 (14.1%)	237 (16.6%)	59 (8.8%)	
Social or mass media	296 (14.1%)	212 (14.8%)	84 (12.5%)	
Family	259 (12.3%)	173 (12.1%)	86 (12.8%)	
Friend	136 (6.5%)	90 (6.3%)	46 (6.8%)	
Nutritionist	16 (0.8%)	6 (0.4%)	10 (1.5%)	
Social worker	8 (0.4%)	4 (0.3%)	4 (0.6%)	
Others	14 (0.7%)	14 (1.0%)	0	
**Enablers of exercise**				0.214
Encouragement from physician	1279 (19.8%)	964 (19.5%)	315 (20.6%)	
Encouragement from nurse	1284 (19.9%)	974 (19.7%)	310 (20.3%)	
Encouragement from family, relatives, or friends	1170 (18.1%)	888 (18.0%)	282 (18.5%)	
Participation to specialized exercise program	974 (15.1%)	765 (15.5%)	209 (13.7%)	
Encouragement from other dialysis patients	912 (14.1%)	682 (13.8%)	230 (15.1%)	
Exercise facility	844 (13.1%)	663 (13.4%)	181 (11.9%)	
**Benefits from the exercise**				< 0.001
Mood	600 (17.3%)	468 (17.3%)	132 (17.2%)	
Strength	595 (17.1%)	460 (17.0%)	135 (17.6%)	
Overall vitality	531 (15.3%)	403 (14.9%)	128 (16.7%)	
Sleeping	459 (13.2%)	345 (12.7%)	114 (14.8%)	
Confidence	397 (11.4%)	312 (11.5%)	85 (11.1%)	
Blood pressure	321 (9.2%)	243 (9.0%)	78 (10.2%)	
Do things what the patient want for yourself	296 (8.5%)	233 (8.6%)	63 (8.2%)	
Muscle cramping	152 (4.4%)	124 (4.6%)	28 (3.6%)	
Intradialytic hypotension	103 (3.0%)	102 (3.8%)	–	
Others	24 (0.7%)	20 (0.7%)	4 (0.5%)	
**Barriers to exercise**				0.170
Poor motivation	301 (25.0%)	236 (24.4%)	65 (27.2%)	
Fatigue	287 (23.8%)	218 (22.6%)	69 (28.9%)	
Lack of time to exercise	186 (15.4%)	150 (15.5%)	36 (15.1%)	
Pain	183 (15.2%)	151 (15.6%)	32 (13.4%)	
Uncertain on how to exercise	72 (6.0%)	66 (6.8%)	6 (2.5%)	
Depressive mood	62 (5.1%)	50 (5.2%)	12 (5.0%)	
Fear of injury	49 (4.1%)	42 (4.3%)	7 (2.9%)	
Lack of equipment or place to exercise	41 (3.4%)	34 (3.5%)	7 (2.9%)	
Unsure about the importance of exercise	24 (2.0%)	19 (2.0%)	5 (2.1%)	

Data are expressed as numbers (%). *P *values were tested using Pearson’s χ^2^ or Fisher’s exact test.

With regard to individuals providing education on the importance of exercise, the proportion of nurses was greater in PD patients than in HD patients. The proportion of other dialysis patients was greater in HD patients than in PD patients. For benefits from exercise, the proportion of sleep was greater in PD patients than in HD patients. There were no significant differences in the number of enablers of exercise or barriers to exercise between the two dialysis modalities.

### Comparison of HRQoL scale scores according to physical activity

In HD patients, all SF-36 scales and symptoms/problems, cognitive function, and QSI scores among kidney disease-specific scales were greater in patients with high physical activity than in those without high physical activity (Table 3). In PD patients, PF, BP, and PCS in SF-6 scales were greater in patients with high physical activity than in those without high physical activity. Overall improvement of HRQoL scale scores according to physical activity was more prominent in HD patients than in PD patients.

**Table 3:** Comparison of quality of life scale scores according to physical activity.

	Hemodialysis			Peritoneal dialysis		
	Non-high PA (n = 1107)	High PA (n = 140)	*P* value	Non-high PA (n = 344)	High PA (n = 20)	*P* value
**SF-6 scale**						
PF	80 (30)	90 (10)	< 0.001	80 (30)	90 (10)	< 0.001
RP	100 (75)	100 (25)	< 0.001	75 (75)	100 (50)	0.070
BP	88 (42)	100 (22)	< 0.001	80 (42)	100 (30)	0.046
GH	45 (30)	55 (35)	< 0.001	40 (30)	40 (22)	0.736
VT	45 (25)	50 (24)	< 0.001	45 (25)	50 (24)	0.076
SF	88 (37)	88 (25)	0.017	75 (37)	75 (34)	0.919
RE	100 (67)	100 (0)	0.004	100 (100)	100 (25)	0.054
MH	60 (28)	64 (20)	0.001	60 (24)	60 (20)	0.432
OHR	25 (25)	50 (25)	< 0.001	25 (25)	25 (25)	0.449
PCS	65.0 (32.4)	75.0 (20.9)	< 0.001	62.6 (29.9)	72.0 (15.5)	0.021
MCS	63.2 (29.8)	70.7 (20.7)	< 0.001	59.3 (30.3)	65.6 (20.3)	0.182
**KD-specific scale**						
Sx	83 (19)	88 (15)	< 0.001	81 (23)	84 (18)	0.501
KD effects	75 (29)	78 (22)	0.113	78 (25)	76 (24)	0.950
KD burden	31 (37)	31 (43)	0.135	31 (43)	31 (47)	0.434
Work status	0 (50)	0 (50)	0.109	0 (50)	0 (50)	0.607
Cognitive function	93 (20)	95 (13)	0.044	93.0 (20)	93 (20)	0.571
QSI	80 (33)	87 (20)	0.004	73 (33)	73 (20)	0.727
Sexual function	88 (50)	88 (25)	0.610	88 (50)	81 (78)	0.713
Sleep	65 (30)	68 (32)	0.403	65 (28)	64 (30)	0.937
Social support	67 (50)	67 (34)	0.349	67 (33)	67 (33)	0.810
Patient satisfaction	67 (33)	67 (33)	0.518	67 (33)	67(29)	0.934
DSE	88 (25)	100 (25)	0.112	100 (25)	100 (25)	0.498

Data are expressed as median (interquartile range). Comparisons between MET < 600 and ≥ 600 were tested using Kolmogorov–Smirnov *z* test.

PA, physical activity; SF, short form; MET, metabolic equivalent of task; PA, physical activity; PF, physical functioning; RP, role limitations due to physical health problems; BP, bodily pain; GH, general health; VT, vitality; SF, social functioning; RE, role limitations due to emotional problems; MH, mental health; OHR, overall health rating; PCS, physical component scale; MCS, mental component scale; KD, kidney disease; Sx, symptom/problems; QSI, quality of social interaction; DSE, dialysis staff encouragement.

## Discussion

Our multicenter cohort study enrolled only relatively stable dialysis patients. We divided patients into two groups according to the dialysis modality. First, the modality per se can be an inherent confounding factor for physical activity or understanding of the importance of exercise. Second, we want to evaluate the association between dialysis modality and physical activity or exercise-related factors. The intensity of physical activity did not differ between the two modalities. Most patients performed low- to moderate-intensity physical activities. The time of physical activity was longer in HD patients than in PD patients, which resulted in greater MET values and the number of high physical activity. Dialysis staff were significantly important for the improvement of exercise in both HD and PD patients, but the importance of dialysis staff was considerably greater in PD patients. The most important barriers to exercise were poor motivation and fatigue in both modalities. HD patients exhibited greater differences in HRQoL scales across the two physical activity levels, compared to PD patients.

In our study, more PD patients recognized the importance of exercise than HD patients. However, HD patients had more time for physical activity than PD patients, but the intensity of physical activity was similar in both modalities. Clinical practitioners or patients may misunderstand that HD patients consume more time for dialysis than PD patients. However, in Korea, most HD patients have undergone HD in a dialysis facility adjacent to their home or workplace, and a number of PD patients have selected the PD modality due to limitation of access to a dialysis facility such as a distant HD facility^19^. Regarding the time for dialysate exchanges or movement to a PD facility, HD patients may not spend much time for dialysis compared to PD patients. Therefore, lack of time as a barrier to exercise was similar in both modalities at approximately 15%, and lack of an exercise facility or program was not considered an important barrier. The most important barriers to exercise and enablers of exercise were poor motivation and encouragement from dialysis staff, respectively. These findings reveal that frequent visits to a dialysis facility or communication with dialysis staff would be of merit to HD patients, which led to a greater proportion of high physical activities or MET values in HD patients than in PD patients. In addition, poor motivation for exercise in PD patients can be improved by proper and sustained education by dialysis staff on the importance of exercise. The association between physical activity and traveling method or distance to the HD facility is a very interesting issue. Traveling method (walking or car) or distance to the HD facility can be associated with physical activity, and physical activity can also influence the selection of dialysis modality, HD facility, or the traveling method. However, our study did not include the data regarding traveling method or interval between home or workplace and HD facility. Additional studies using these data would be useful in identifying the association between these variables.

Delgado et al. investigated the recognition to exercise in 100 HD patients^20^. They compared two groups, one with low and the other with high physical activity. Although the most commonly reported barrier to exercise was fatigue, statistical significance was not achieved using multivariate analysis. Lack of motivation, many medical problems, and not having enough time, a safe place to exercise, or an exercise partner were significantly associated with low physical activity. An integrative review study was performed using data from 14 studies regarding data for barriers to exercise in chronic kidney disease^21^. Although questionnaires regarding barrier to exercise were inconsistent and the patients’ reports on barriers were complex and diverse, the most frequently identified barrier was fatigue or lack of energy. Further, studies evaluated barriers to exercise or physical activity, but certain limitations remained. First, most of the studies had a relatively small sample size between 22 and 269 (the median sample size from 14 studies was 88). Second, most studies focused on HD patients alone. The 12 of 13 studies using dialysis patients were performed using HD patients alone and a total sample size of 1 study using both HD and PD patients consisted of only 78 responses (including 19 from PD patients). Third, 7 of 14 studies were carried out on multiple ethnicities, 2 studies evaluated only a single ethnicity, and 5 studies did not present ethinicity as a variable. Although multiple ethnicities might be useful in studies in terms of generalizability, this may be confounding element in small sample size studies. Our study enrolled both HD and PD patients and had both a large sample size and a single ethnicity (only Asian dialysis patients, including 1,247 HD and 364 PD patients). Our results showed that poor motivation and fatigue were most common barriers to exercise in HD patients (similar results compared to previous results) and same trends were also observed from PD patients.

Although there are few high-quality studies regarding the association between exercise and HRQoL scales in dialysis patients, many clinical practitioners suggest that exercise can improve both physical health and MH^22–25^. In our study, almost all scales associated with physical health and MH were greater in HD patients with high physical activity than in those without high physical activity. Dialysis patients also understood the impact of exercise on mood, strength, or VT. For the kidney disease-specific scale, symptoms/problems, cognitive function, and QSI were greater in HD patients with high physical activity than in those without high physical activity. However, there were no statistically significant differences in PD patients with high physical activity compared to in those without high physical activity (except body pain). Two issues may be associated with this non-difference. Sustained peritoneal dialysate in the abdomen can attenuate the impact of physical activity on some physical health/MH scales or the kidney disease-specific scales, and a small number of PD patients on high physical activity also led to statistical non-significance. In addition, disability, frailty, fall, or exhaustion was greater in HD patients without high physical activity than in those with high physical activity. Moreover, in PD patients, frailty alone differed between the two groups. However, trends for disability, fall, or exhaustion were similar with the results from HD patients.

In our study, clinical symptom was not an important barrier to exercise. Moorman et al. showed that the barrier to exercise was excessive tiredness or weakness or shortness of breath^26^. In our study, all dialysis patients were ambulatory and medically stable. Therefore, only a few patients had difficulty in exercising due to a medical problem such as shortness of breath, except pain.

The present study had a few limitations. First, it had a retrospective design and used data on participants enrolled in a previous study^8^. In addition, our study was analyzed using data collected at approximately 10 years ago. Many factors, including patients’ characteristics, social, or environmental conditions at onset of data would be different compared to those using recent patients, which may lead to different results. In addition, social, educational, cultural, and environmental factors can influence outcomes related to physical activity, quality of life, and awareness of the need for exercise. However, we did not perform analyses using these data. Second, the present study did not include data from physical performance measurements as an accurate indicator, and the presence of physical activity and understanding of the importance of exercise were evaluated using questionnaires alone. In addition, our data did not include the data regarding bicarbonate, Kt/Vurea, and dialysis treatment adherence and we did not perform a multivariate analysis including these variables. Our results were evaluated without adjusting these variables as possible confounding factors. Current guidelines recommend Kt/Vurea ≥ 1.2 for HD and weekly Kt/Vurea ≥ 1.7 for PD^27, 28^. Previous data from a representative sample of Koreans showed that the proportion of patients with inadequate HD was 8.5%^29^. Mean weekly Kt/Vurea for PD was 1.9 ± 0.8 in Korea and a significant portion of PD would be adequate^2^. Most of the patients from our study may be undergoing adequate dialysis and these may attenuate the effects on physical disability or activity resulting from significantly inadequate dialysis. Third, our study, as a cross-sectional analysis, did not evaluate the causal relationship between physical activity and outcome measurements such as HRQoL scales, disability, frailty, fall, or exhaustion. Fourth, high physical activity was defined as participants ≥ 600 MET, and the number of participants with MET values ≥ 3000, which was classified as a high amount of physical activity, was only 5 (0.4%) in HD patients and 0 in PD patients. Therefore, we did not evaluate the impact of the amount of physical activity. Fifth, exhaustion in our study was defined using two of 20 questions from the CES-D scale for the diagnosis of depression. These 2 questions were classified as indicators of somatic activity^30^. Evaluation of exhaustion using two questions saves time and is simple. These are originally derived from a questionnaire for the diagnosis of a depressive mood and may not accurately reflect an experience of physical exhaustion. However, Vestergaard et al. evaluated the presence of exhaustion using two questions and observations of physical functioning^31^. They demonstrated that there was a positive association between the presence of exhaustion and physical activities, including handgrip strength, gait speed, disability, and a short physical performance battery. In addition, Fried et al. used these two questions as a component in their diagnosis of frailty^14^. A prospective study including additional parameters such as physical performance measurements and interventions for exercise is warranted to overcome these limitations.

In conclusion, our study showed that the barrier to exercise and the enablers of exercise were poor motivation/fatigue and encouragement from dialysis staff, respectively. The frequent contact with medical staff in a HD facility may be associated with a greater amount of high physical activity in HD patients than in PD patients.

## Supplementary Information

Supplementary Tables.
