# Supplementary material for: Comparisons of physical activity and understanding of the importance of exercise according to dialysis modality in maintenance dialysis patients
Source: Sci Rep. 2021 Nov 2;11:21487. doi: 10.1038/s41598-021-00924-0 (PMC8564543; doi:10.1038/s41598-021-00924-0)
Supplement: Supplementary file 1 — Supplementary Tables. [file 41598_2021_924_MOESM1_ESM.docx]

**Comparisons of physical activity and understanding of the importance of exercise according to dialysis modality in maintenance dialysis patients**

Jun Chul Kim^1^, Jun Young Do^2^, and Seok Hui Kang^2^

^1^Division of Nephrology, Department of Internal Medicine, CHA Gumi Medical Center, CHA University, Gumi, Republic of Korea

^2^Division of Nephrology, Department of Internal Medicine, College of Medicine, Yeungnam University, Daegu, Republic of Korea

**Supplementary Information**

Table S1: Linear regression analysis of MET value according to variables.

Table S2. Logistic regression analysis of high physical activity according to variables.

Table S3. Comparison of disability, frailty, fall, or exhaustion according to physical activity.

**Table S1. Linear regression analysis of MET value according to variables**

|  | **Univariate** | | **Multivariate** | |
| --- | --- | --- | --- | --- |
|  | St-β (SE) | *P*-value | St-β (SE) | *P*-value |
| Age (ref: < 65 years) | –0.10 (21.20) | <0.001 | –0.11 (25.75) | 0.001 |
| Sex (ref: men) | –0.09 (18.68) | <0.001 | –0.10 (22.64) | 0.004 |
| BMI (increase 1 kg/m^2^) | –0.02 (2.90) | 0.458 | –0.07 (3.62) | 0.048 |
| DM (ref: non-DM) | –0.05 (19.03) | 0.059 | – | – |
| CAD (ref: non-CAD) | –0.04 (25.54) | 0.110 | – | – |
| CVD (ref: non-CVD) | –0.01 (32.65) | 0.663 | – | – |
| Serum albumin (increase in 1 g/dL) | 0.05 (24.28) | 0.033 | – | – |
| BUN (increase 1 mg/dL) | 0.10 (0.62) | <0.001 | 0.09 (0.78) | 0.010 |
| Creatinine (increase 1 mg/dL) | 0.12 (3.09) | <0.001 | – | – |
| Total cholesterol (increase 1 mg/dL) | –0.00 (0.25) | 0.863 | – | – |
| Serum potassium (increase 1 mEq/L) | 0.06 (12.25) | 0.028 | – | – |
| i-PTH (increase 1 pg/mL) | –0.04 (0.03) | 0.141 | – | – |
| hs-CRP (increase 1 mg/dL) | –0.01 (6.97) | 0.678 | – | – |
| Dialysis modality (ref: PD) | 0.07 (22.22) | 0.003 | 0.08 (24.96) | 0.034 |

Multivariate analysis was performed using forward mode and adjusted for age, sex, BMI, DM, CAD, CVD, serum albumin, BUN, creatinine, total cholesterol, i-PTH, hs-CRP, serum potassium, and dialysis modality.

Abbreviations: MET, metabolic equivalent of task; St-β, standardized beta; SE, standard error; BMI, body mass index; DM, diabetes mellitus; CAD, coronary artery disease; CVD, cerebrovascular disease; BUN, blood urea nitrogen; i-PTH, intact parathyroid hormone; hs-CRP, high sensitivity C-reactive protein; PD, peritoneal dialysis.

**Table S2. Logistic regression analysis of high physical activity according to variables**

|  | **Univariate** | | **Model 1** | | **Model 2** | |
| --- | --- | --- | --- | --- | --- | --- |
|  | OR (95% CI) | *P*-value | OR (95% CI) | *P*-value | OR (95% CI) | *P*-value |
| Age (ref: < 65 years) | 0.38 (0.24–0.62) | <0.001 | 0.45 (0.22–0.89) | 0.022 | 0.43 (0.22–0.87) | 0.019 |
| Sex (ref: men) | 0.64 (0.45–0.89) | 0.009 | – | – | – | – |
| BMI (increase 1 kg/m^2^) | 0.99 (0.94–1.04) | 0.754 | – | – | – | – |
| DM (ref: non-DM) | 0.69 (0.49–0.98) | 0.037 | – | – | – | – |
| CAD (ref: non-CAD) | 0.53 (0.30–0.91) | 0.021 | – | – | – | – |
| CVD (ref: non-CVD) | 0.97 (0.55–1.74) | 0.930 | – | – | – | – |
| Serum albumin (ref: < 3.5 g/dL) | 1.66 (1.15–2.40) | 0.007 | 2.49 (1.02–6.09) | 0.045 | 2.51 (1.03–6.13) | 0.043 |
| BUN (increase 1 mg/dL) | 1.02 (1.01–1.03) | <0.001 | – | – | – | – |
| Creatinine (increase 1 mg/dL) | 1.13 (1.07–1.19) | <0.001 | 1.16 (1.07–1.26) | <0.001 | 1.16 (1.07–1.26) | <0.001 |
| Total cholesterol (increase 1 mg/dL) | 1.00 (1.00–1.01) | 0.526 | 1.01 (1.00–1.01) | 0.031 | 1.01 (1.00–1.01) | 0.029 |
| Serum potassium (increase 1 mEq/L) | 1.26 (1.01–1.56) | 0.037 | – | – | – | – |
| i-PTH (pg/mL) | 1.00 (1.00–1.00) | 0.569 | – | – | – | – |
| hs-CRP (increase 1 mg/dL) | 0.95 (0.81–1.12) | 0.536 | – | – | – | – |
| Dialysis modality (ref: PD) | 2.18 (1.34–3.53) | 0.002 | 3.01 (1.64–5.54) | <0.001 | – | – |
| Type of dialysis |  |  |  |  |  |  |
| AVF (ref: PD) | 2.25 (1.38–3.66) | 0.001 | – | – | 2.94 (1.59–5.44) | 0.001 |
| AVG (ref: PD) | 1.82 (0.95–3.49) | 0.074 | – | – | 3.74 (1.48–9.47) | 0.005 |
| AVG (ref: AVF in HD) | 0.81 (0.49–1.35) | 0.414 | – | – | 1.27 (0.58–2.80) | 0.549 |

Multivariate logistic regression analysis was performed using the forward conditional method.

Model 1 was adjusted for age, sex, BMI, DM, CAD, CVD, serum albumin, BUN, creatinine, total cholesterol, i-PTH, hs-CRP, serum potassium, and dialysis modality. Model 2 was adjusted for age, sex, BMI, DM, CAD, CVD, serum albumin, BUN, creatinine, total cholesterol, i-PTH, hs-CRP, serum potassium, and type of dialysis.

Abbreviations: OR, odds ratio; CI, confidence interval; BMI, body mass index; DM, diabetes mellitus; CAD, coronary artery disease; CVD, cerebrovascular disease; BUN, blood urea nitrogen; i-PTH, intact parathyroid hormone; hs-CRP, high sensitivity C-reactive protein; PD, peritoneal dialysis; AVF, arteriovenous fistula; AVG, arteriovenous graft; HD, hemodialysis.

**Table S3. Comparison of disability, frailty, fall, or exhaustion according to physical activity**

|  | **Hemodialysis** | | |  | **Peritoneal dialysis** | | |  |
| --- | --- | --- | --- | --- | --- | --- | --- | --- |
|  | **Non-high PA**  **(n = 1,107)** | **High PA**  **(n = 140)** | ***P*** |  | **Non-high PA**  **(n = 344)** | **High PA**  **(n = 20)** | ***P*** | |
| Disability | 182 (16.4%) | 13 (9.3%) | 0.028 |  | 106 (30.8%) | 3 (15.0%) | 0.133 | |
| Frailty | 408 (36.9%) | 13 (9.3%) | <0.001 |  | 136 (39.5%) | 0 | <0.001 | |
| Fall | 205 (18.5%) | 20 (14.3%) | 0.220 |  | 59 (17.2%) | 1 (5.0%) | 0.155 | |
| Exhaustion | 323 (29.2%) | 24 (17.1%) | 0.003 |  | 120 (34.9%) | 4 (20.0%) | 0.172 | |

Data are expressed as numbers (percentage). Comparisons between non-high PA and high PA were tested using Chi-square test.

Abbreviations: PA, physical activity.
